# Author Correction: Clinical phenotypes and outcomes associated with SARS-CoV-2 variant Omicron in critically ill French patients with COVID-19

**DOI:** 10.1038/s41467-022-34575-0

**Published:** 2022-12-07

**Authors:** Nicolas de Prost, Etienne Audureau, Nicholas Heming, Elyanne Gault, Tài Pham, Amal Chaghouri, Nina de Montmollin, Guillaume Voiriot, Laurence Morand-Joubert, Adrien Joseph, Marie-Laure Chaix, Sébastien Préau, Raphaël Favory, Aurélie Guigon, Charles-Edouard Luyt, Sonia Burrel, Julien Mayaux, Stéphane Marot, Damien Roux, Diane Descamps, Sylvie Meireles, Frédéric Pène, Flore Rozenberg, Damien Contou, Amandine Henry, Stéphane Gaudry, Ségolène Brichler, Jean-François Timsit, Antoine Kimmoun, Cédric Hartard, Louise-Marie Jandeaux, Samira Fafi-Kremer, Paul Gabarre, Malo Emery, Claudio Garcia-Sanchez, Sébastien Jochmans, Aurélia Pitsch, Djillali Annane, Elie Azoulay, Armand Mekontso Dessap, Christophe Rodriguez, Jean-Michel Pawlotsky, Slim Fourati

**Affiliations:** 1 Médecine Intensive Réanimation, Hôpitaux Universitaires Henri Mondor, Assistance Publique—Hôpitaux de Paris (AP-HP), Créteil, France; 2 Groupe de Recherche Clinique CARMAS, Université Paris-Est-Créteil (UPEC), Créteil, France; 3 Université Paris-Est-Créteil (UPEC), Créteil, France; 4 Department of Public Health, Hôpitaux Universitaires Henri Mondor, Assistance Publique—Hôpitaux de Paris (AP-HP), Créteil, France; 5 IMRB INSERM U955, Team CEpiA, Créteil, France; 6 Médecine Intensive Réanimation, Hôpital Raymond Poincaré, Assistance Publique—Hôpitaux de Paris (AP-HP), Garches, France; 7 Laboratoire de Virologie, Hôpital Ambroise Paré, Assistance Publique—Hôpitaux de Paris (AP-HP), Boulogne, France; 8 Service de Médecine Intensive-Réanimation, Assistance Publique—Hôpitaux de Paris, Hôpital de Bicêtre, DMU 4 CORREVE Maladies du Cœur et des Vaisseaux, FHU Sepsis, Le Kremlin-Bicêtre, France; 9 Inserm U1018, Equipe d’Epidémiologie respiratoire intégrative, CESP, 94807 Villejuif, France; 10 Laboratoire de Virologie, Hôpital Paul Brousse, Assistance Publique—Hôpitaux de Paris, Villejuif, France; 11 Sorbonne Université, Centre de Recherche Saint-Antoine INSERM, Médecine Intensive Réanimation, Hôpital Tenon, Assistance Publique—Hôpitaux de Paris, Paris, France; 12 Sorbonne Université, INSERM, Institut Pierre Louis d’Epidémiologie et de Santé Publique, Paris, France; 13 Laboratoire de virologie, Hôpital Saint-Antoine, Assistance Publique—Hôpitaux de Paris, F-75012 Paris, France; 14 Médecine Intensive Réanimation, Hôpital Saint-Louis, Assistance Publique—Hôpitaux de Paris, Paris, France; 15 Université de Paris, Inserm HIPI, F-75010 Paris, France; 16 Laboratoire de Virologie, Hôpital Saint-Louis, Assistance Publique—Hôpitaux de Paris, F-75010 Paris, France; 17 U1167—RID-AGE Facteurs de Risque et Déterminants Moléculaires des Maladies Liées au Vieillissement, University Lille, Inserm, CHU Lille, Institut Pasteur de Lille, F-59000 Lille, France; 18 Service de virologie, CHU de Lille, F-59000 Lille, France; 19 Sorbonne Université, Assistance Publique—Hôpitaux de Paris, Hôpital Pitié–Salpêtrière, Médecine Intensive Réanimation, Paris, France; 20 INSERM UMRS_1166-iCAN, Institute of Cardiometabolism and Nutrition, Paris, France; 21 Département de Virologie, Hôpital Pitié–Salpêtrière, Assistance Publique-Hôpitaux de Paris (AP-HP), Paris, France; 22 Sorbonne Université, Assistance Publique–Hôpitaux de Paris, Hôpital Pitié–Salpêtrière, Médecine Intensive Réanimation, Paris, France; 23 Université de Paris, APHP, Hôpital Louis Mourier, DMU ESPRIT, Service de Médecine Intensive Réanimation, Colombes, France; 24 INSERM U1151, CNRS UMR 8253, Institut Necker-Enfants Malades (INEM), Department of Immunology, Infectiology and Hematology, Paris, France; 25 Université de Paris, IAME INSERM UMR 1137, Service de Virologie, Hôpital Bichat-Claude Bernard, Assistance Publique—Hôpitaux de Paris, Paris, France; 26 Service de Réanimation médico-chirurgicale, Assistance Publique–Hôpitaux de Paris, Hôpital Ambroise Paré, Boulogne, France; 27 Médecine Intensive Réanimation, Hôpital Cochin, Assistance Publique—Hôpitaux de Paris, Paris, France; 28 Laboratoire de Virologie, Hôpital Cochin, Assistance Publique—Hôpitaux de Paris, Paris, France; 29 Service de Réanimation, Hôpital Victor Dupouy, Argenteuil, France; 30 Service de Virologie, Hôpital Victor Dupouy, Argenteuil, France; 31 Service de Réanimation, Hôpital Avicenne, Assistance Publique—Hôpitaux de Paris, Bobigny, France; 32 Laboratoire de Virologie, Hôpital Avicenne, Assistance Publique—Hôpitaux de Paris, Bobigny, France; 33 Service de Médecine Intensive Réanimation, Hôpital Bichat, Assistance Publique—Hôpitaux de Paris, Paris, France; 34 Université de Lorraine, CHRU de Nancy, Médecine Intensive et Réanimation Brabois, Vandœuvre-lès-Nancy, France; 35 INSERM U942 and U1116, F-CRIN-INIC RCT, Vandœuvre-lès-Nancy, France; 36 Service de Virologie, CHRU de Nancy, Vandœuvre-lès-Nancy, France; 37 INSERM (French National Institute of Health and Medical Research), UMR 1260, Regenerative Nanomedicine (RNM), CRBS (Centre de Recherche en Biomédecine de Strasbourg), FMTS (Fédération de Médecine Translationnelle de Strasbourg), University of Strasbourg, Strasbourg, France; 38 Department of Intensive Care (Service de Médecine Intensive - Réanimation), Nouvel Hôpital Civil, Hôpital Universitaire de Strasbourg, Strasbourg, France; 39 Service de Virologie, Nouvel Hôpital Civil, Hôpital Universitaire de Strasbourg, Strasbourg, France; 40 Sorbonne Université, Assistance Publique—Hôpitaux de Paris, Hôpital Saint-Antoine, Médecine Intensive Réanimation, 75571 Paris, Cedex 12, France; 41 Service de Réanimation, Hôpital Saint-Camille, Bry-sur-Marne, France; 42 Laboratoire de Biologie, Hôpital Saint-Camille, Bry-sur-Marne, France; 43 Service de Réanimation Polyvalente, Hôpital Marc Jacquet, Melun, France; 44 Laboratoire de Microbiologie, Hôpital Marc Jacquet, Melun, France; 45 Department of Virology, Hôpitaux Universitaires Henri Mondor, Assistance Publique—Hôpitaux de Paris, Créteil, France; 46 INSERM U955, Team « Viruses, Hepatology, Cancer », Créteil, France

**Keywords:** SARS-CoV-2, Risk factors, Respiratory signs and symptoms

Correction to: *Nature Communications* 10.1038/s41467-022-33801-z, published online 12 October 2022

The original version of this Article contained an error in Fig. 2b, in which “Day 3” was missing in the x-axis label. This has been corrected in both the PDF and HTML versions of the Article.

